# Exchequer Grants and the Public Health

**Published:** 1924-03

**Authors:** 


### EXCHEQUER GRANTS AND THE PUBLIC HEALTH.

The action of the new Minister of Health in removing the restrictions on the development of the grant-aided public health services is not, at first blush, one which those who are enthusiastic for the advancement of the public health might be expected to criticise. It is spectacular, without doubt. The Minister is in a position to announce to his supporters that as soon as he assumed office he took up his pen and with a stroke and a flourish removed the restrictions which were preventing willing authorities from doing all that they wanted to do for weak and suffering humanity. It is not really so simple as all that. The services mainly affected are Maternity and Child Welfare, Tuberculosis, and Venereal Disease. Exchequer grants have been paid in aid of approved expenditure of local authorities on these services on a fifty per cent., or pound for pound, basis in the case of the two first-mentioned services and seventy-five per cent. in the case of venereal disease. The restrictions imposed in the last two years by Ministers of Health have taken the form of rationing the local authorities by imposing for each area a maximum limit of expenditure on which the Exchequer grant would be paid. This would not prevent such authorities from spending on these services, out of local rates, such further sums as they should think fit.

The action of the Minister, as we say, is spectacular, but is it statesmanlike, or in pursuance of a well-considered policy? We must not be understood as suggesting an actual curtailment of expenditure on the services to which we have referred. But the value to the community must inevitably be judged by results, and there is not a public health authority which does not realise that environmental conditions, left undisturbed because of lack of money, are manufacturing fresh cases of disease faster than old cases are being cured or arrested, or are dying out, as the case may be. With a limited public purse it is well to recognise that every pound spent on services designed for the benefit of the individual diminishes the sum available to be spent on environment and the prevention of disease.

In an article which appeared in this Review in October, 1922, by Dr. Wynne, the Medical Officer of Health for Sheffield, "The Wrong Turning in Public Health," with much of which we find ourselves in agreement the writer expressed the view that it was the duty of public health administrators to resist the temptation to yield to popular clamour in favour of purely eleemosynary schemes or methods of advertisement which tend to divert public funds from the prevention of disease to tinkering with disease when it has developed. This is sound doctrine. We are in favour of the maintenance of a wisely-directed public expenditure on the three great services referred to, subject to the grant in the case of venereal disease being put on the fifty per cent. basis, as in the case of the other and more important services. But the extension of Exchequer assistance involved by the order of the new Minister seems to have been undertaken with some precipitancy and either without regard to, or in disregard of, its effect on the other, and more legitimate, field of public health expenditure.

